# A Free Virtual Reality Experience to Prepare Pediatric Patients for Magnetic Resonance Imaging: Cross-Sectional Questionnaire Study

**DOI:** 10.2196/11684

**Published:** 2019-04-18

**Authors:** Jonathan Ashmore, Jerome Di Pietro, Kelly Williams, Euan Stokes, Anna Symons, Martina Smith, Louise Clegg, Cormac McGrath

**Affiliations:** 1 Department of Medical Physics and Bioengineering NHS Highland Inverness United Kingdom; 2 Department of Neuroradiology King's College Hospital London United Kingdom; 3 Centre for Neuroimaging Sciences King's College London London United Kingdom; 4 Faculty of Life Sciences & Medicine King's College London London United Kingdom; 5 King's College Hospital London United Kingdom; 6 South London and the Maudsley NHS Trust London United Kingdom; 7 Radiological Sciences & Imaging Regional Medical Physics Service Belfast Health and Social Care Trust Belfast United Kingdom

**Keywords:** virtual reality, MRI, anxiety

## Abstract

**Background:**

A magnetic resonance image (MRI) is a diagnostic test that requires patients to lie still for prolonged periods within a claustrophobic and noisy environment. This can be difficult for children to tolerate, and often general anesthetic (GA) is required at considerable cost and detriment to patient safety. Virtual reality (VR) is a newly emerging technology that can be implemented at low cost within a health care setting. It has been shown to reduce fear associated with a number of high-anxiety situations and medical procedures.

**Objective:**

The goal of the research was to develop a VR resource to prepare pediatric patients for MRI, helping to reduce anxieties in children undergoing the procedure.

**Methods:**

A freely accessible VR preparation resource was developed to prepare pediatric patients for their upcoming MRI. The resource consists of an app and supporting preparation book and used a series of panoramic 360 degree videos of the entire MRI journey, including footage from within the bore of the scanner. The app, deployed via the Android Play Store and iOS App Store, can be viewed on most mobile phones, allowing a child to experience an MRI in VR using an inexpensive Google Cardboard headset. The app contains 360 degree videos within an animated, interactive VR interface designed for 4 to 12-year-olds. The resource was evaluated as part of a clinical audit on 23 patients (aged 4 to 12 years), and feedback was obtained from 10 staff members. In 5 patients, the resource was evaluated as a tool to prepare patients for an awake MRI who otherwise were booked to have an MRI under GA.

**Results:**

The VR preparation resource has been successfully implemented at 3 UK institutions. Of the 23 patients surveyed, on a scale of 1 to 10, the VR resource was rated with a median score of 8.5 for enjoyment, 8 for helpfulness, and 10 for ease of use. All patients agreed that it made them feel more positive about their MRI, and all suggested they would recommend the resource to other children. When considering their experiences using the resource with pediatric patients, on a scale of 1 to 10, the staff members rated the VR resource a median score of 8.5 for enjoyment, 9 for helpfulness, and 9 for ease of use. All staff believed it could help prepare children for an awake MRI, thus avoiding GA. A successful awake MRI was achieved in 4 of the 5 children for whom routine care would have resulted in an MRI under GA.

**Conclusions:**

Our VR resource has the potential to relieve anxieties and better prepare patients for an awake MRI. The resource has potential to avoid GA through educating the child about the MRI process.

## Introduction

The claustrophobic and noisy environment of magnetic resonance imaging (MRI) can be difficult for patients to tolerate and at times can lead to patient movement during the scan or even to the scan being aborted. If the resultant images are undiagnostic, this may require the patient to be rescanned under sedation or general anesthesia (GA). Children represent a particularly sensitive patient group to such effects where the rescan rate and need for GA is considerably higher than in adults. In a recent study it was shown that the use of GA in pediatric MRI has risen from 21% to 28% over 3 years, an effect attributed to the increased use of 3 Tesla MRI systems, which produce superior image quality but have an associated increased sensitivity to patient motion [1].

Despite this increase, previous work has shown that appropriate preparation can successfully reduce the need for anesthesia in younger patients. In one study, through the effective use of multiple preparation resources, anesthesia rates were reduced from 47% to 27% [2]. A variety of methods have been reported to prepare children for MRI including the use of informational videos [3], mock scanners [4], play tunnels, vibrating mats with MRI scanner audio simulations [5], and the use of small scale models for demonstrating the MRI procedure to children [6]. Having a process to avoid anesthesia is also preferred among parents [7,8].

There are numerous benefits to both the patient and the institution in avoiding an MRI scan with GA. With an awake MRI, the risks of possible adverse reaction to GA or other forms of sedation are removed, which although unlikely can still occur [9]. Research has demonstrated that anesthesia can be highly anxiety-provoking for children and parents [10] because there is the need to fast prior to GA and a recovery period requiring them to remain in clinical care for typically 1 to 2 hours. Because the GA requires medical intervention, there are also significant costs associated with the equipment and clinical team required to administer the procedure.

In recent years, the use of virtual reality (VR) in medicine has undergone rapid growth with applications in surgery [11], teaching and training [12], anxiety reduction for medical procedures [13-15], and treatment for a variety of diseases [16-18]. This increase in popularity has been driven by the development of consumer-level VR headsets that use a standard mobile phone (eg, Google Cardboard and Daydream View, Google LLC; Gear VR, Samsung Electronics Co Ltd) or a PC (eg, Oculus Rift, Facebook Technologies LLC; Vive, HTC Corporation) for their computer processing. In particular, the Google Cardboard format has potential for large-scale distribution, since most modern mobile phones have the technology to be incorporated into a simple, cheap headset to display content in the VR format. Distribution of content is now easily facilitated via YouTube, which has native cardboard compatibility, or Google Play for Android devices and the App Store for iOS devices. Creating VR content is also now more achievable with the availability of consumer-level 360° cameras that allow amateur film makers to create and easily distribute 360° footage for viewing within a VR headset.

In this paper, we report on a freely accessible resource that uses 360° video footage to acclimatize a pediatric patient for an upcoming MRI using VR. The resource was created as a tool to help lower anxiety associated with MRI, reduce motion-related image artifact, and decrease the need for GA by increasing awake MRI compliance.

## Methods

### The Virtual Reality Magnetic Resonance Imaging Journey

The VR resource was developed to facilitate preparation of pediatric patients for MRI in a number of settings. First, the resource was developed to be used by health play specialists in their role supporting extremely anxious patients. Second, the resource was to be available to patients with upcoming MRI appointments. Information was included in their appointment letter regarding how to download the resource. By using the resource at home, the child would be in a familiar and safe environment that may better facilitate learning and discussion. Third, we used the resource in the MRI waiting area for anxious patients and parents prior to their MRI. Finally, the resource was used during the anesthetic preassessment process several weeks prior to a patient’s MRI under GA. In this setting, the resource may help guide the decision regarding whether an awake MRI is achievable. All these use cases were suggested by health play specialists and radiographers whose daily role it was to prepare patients for MRI. Both groups had active involvement in the design of the resource and informed the development from the beginning using relevant insights from their roles in health care. They highlighted the importance of showing the pathway that a child undergoes when having an MRI, specifically focusing on key parts of the journey to help the patient to understand and experience each aspect of their upcoming scan. These included (1) arriving at the reception and the waiting area, (2) participating in the MRI safety screening process, (3) highlighting where the radiographer sits during the scan, (4) introducing the children to the scan room, (5) allowing them to experience being in the scanner, and (6) saying goodbye after their scan. All elements were captured via 360° video and incorporated into the resource. The use of 360° video rather than computer-generated content facilitated scalability to other sites that could capture custom footage for their own bespoke version. The resource was aimed at children between ages 4 and 12 years, an age group where appropriate preparation can greatly increase the chance that the child will tolerate and lie still for their scan. The resource was deployed as a free downloadable app and a supporting preparation book. Having the two helped facilitate the different interactions required by different patients and provided a choice of resource for the patient to access. The aim of the app was to produce a continually immersive VR experience of the entire MRI journey, easily accessible for home use. The preparation book helped facilitate a greater interaction between clinical staff and anxious patients. Rather than a continual immersive experience, it fostered discussion outside of VR and, when appropriate, the individual 360° videos could be displayed to the patient using the VR headset.

Incorporating the entire MRI journey into the resource was felt to be important since all elements in the journey can induce anxiety for a child. It was hoped that the virtual experience would better allow children to understand their role and the roles of others when coming for an MRI, helping them to feel involved with their own care. It was designed to highlight the expectation of them in the process and facilitated rehearsal of the procedure. The resource aimed to address common questions asked by children coming for an MRI (eg, concerns that the scanner may touch or hurt them). There was a focus to keep the content positive while realistic, using child-friendly terminology (eg, calling the head coil, used to acquire the MRI images, a helmet) together with highlighting that children could watch their own DVD during the scan.

All persons who appeared in the videos or photos provided consent for their footage and pictures to be used according to our standard hospital policies.

### Capturing 360° Footage

The 360° degree video footage was captured on a 2016 Gear 360 camera (Samsung Electronics Co Ltd) controlled remotely using a Samsung Galaxy S7 mobile phone. For all footage outside the scanner, the camera was positioned using a monopod with tripod stand base at a typical six-year-old child eye height. This allowed for the feeling of being present from the child’s perspective when the videos were viewed within a VR headset. Short video segments were captured where the radiographer and parent actors played out the role as though the child were attending for their scan. We tried to keep the footage length to a minimum time (ideally less than 1 minute) to ensure it remained engaging for the intended young audience but still informative. Initial attempts were made to move the camera during the journey, but it was found that this movement when displayed in a VR headset created a feeling of nausea and this idea was abandoned. Instead stationary footage was taken from key steps of the journey, and the patient was “teleported” via interactions with the app or preparation book from one area of the radiology department to subsequent areas as they progressed along their journey.

The camera was found to function correctly within an operating 1.5T MRI scanner. Scanners tested included the Magnetom Aera 1.5T (Siemens Healthineers), Signa HDx (GE Healthcare), and the Ingenia and Achieva 1.5T (Koninklijke Philips NV). An initial investigation was undertaken by the Magnetic Resonance Safety Expert (MRSE; JA, CMcG) to assess the safety of the camera within the MRI system, and footage was obtained from within the scanner under direct supervision of the MRSE. It was found that the camera did contain some ferromagnetic components which led to mild attractive forces. The weight of the camera mostly overcame these forces except at the location of highest spatial gradient (at the flaring of the MRI scanner bore). To ensure the camera did not become a projectile, it was taped into place on top of a standard phantom that was supplied with the MRI system.

**Figure 1:**
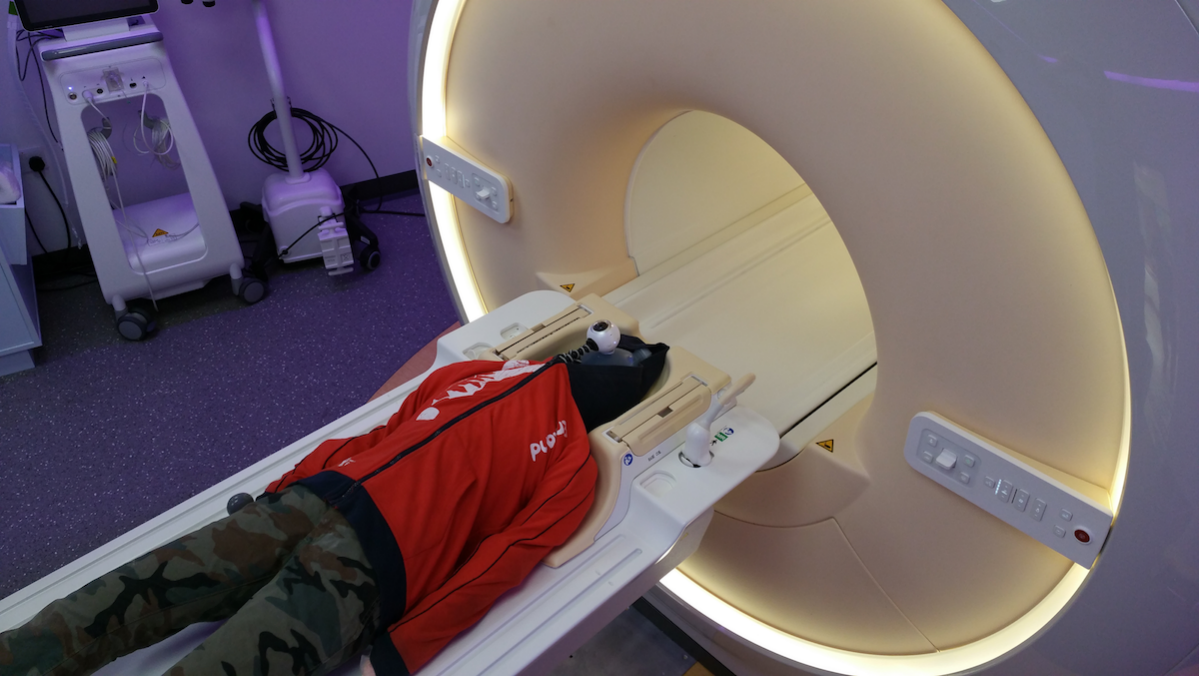
Setup for filming 360 degree video within an operating magnetic resonance imaging scanner.

By fixing the camera onto the phantom, we ensured the scanner would receive a measurable signal allowing it to operate as normal. To provide the illusion of a body for the camera footage, an inflatable mannequin with the head sealed down was dressed in child’s clothing, and the camera, attached to the phantom, acted as the head of the mannequin (see Figure 1).

The 360° footage was successfully obtained during scanner operation with the camera controlled via the Samsung Galaxy S7 phone from within the scan room. Footage was acquired for the localizer and approximately 1 minute of a spoiled gradient echo sequence. In this sequence, the flip angle was reduced to 1 degree to minimize specific absorption rate exposure to the camera. Initially, the camera showed no detrimental effects while in the scanner or after being removed. However, after approximately 10 sessions of capturing footage, the camera started to automatically shut down during footage acquisition within the scanner. At first it was thought the scanner was causing the camera battery to rapidly drain. However, when the camera was removed from the high magnetic field of the scanner, it would operate again as normal, and to the best of our knowledge there was seemingly limited battery drain and no permanent damage. We found it was possible to maximize the camera operating time for filming in the scanner by ensuring the camera was fully charged prior to filming (typically the maximum filming time would be 2 minutes).

Footage was downloaded to the mobile phone, and the Samsung Gear 360 app was used to stitch the two images from the camera sensors into a single equirectangular image. This stitched image was then downloaded to a MacBook Pro for video postprocessing using the package iMovie v10.1.2 (Apple Inc). Editing steps included cropping the video timeline to remove unwanted footage from the start and end. Volume levels were modified to ensure consistency across the clips, and the volume of the operating scanner was reduced such that it was realistic to what a patient would experience during their scan. Audio from the DVD shown during the scan was not captured and instead was layered over the video during postprocessing to ensure the volume was appropriately set. The editing package iMovie outputs video with a 16:9 aspect ratio, and this was subsequently cropped to a 2:1 aspect ratio using the open-source video transcoder HandBrake (360° video has a 2:1 aspect ratio because the field of view is 360° along the horizontal direction by 180° along the vertical). The final 360° video was injected with metadata using the 360 Video Metadata Tool provided by Google.

### The App

The app was created within the Unity development environment (Unity Technologies) and was deployed to both Android and iOS devices via hosting on Google Play and the App Store for free download. The app was designed with a VR format suitable for Google Cardboard–compatible headsets (VR mode) and for use in a standard non-VR format (tablet mode) for patients who did not have a Google Cardboard headset.

The app was designed to be fun and interactive, allowing the child to view the 360° videos in any order or repeat a single video. To maintain a child’s interest, the videos were embedded and accessed via a stylized cartoon environment. A virtual character chosen to match the target patient age, complete with voiceover, was created as a conscious design decision to enable engagement with the target audience.

In tablet mode, swipe and touch interactions were used for selecting and navigating within the 360° videos. In VR mode, the gaze selection method and standard Cardboard buttons were enabled for selecting, pausing, and exiting videos. An initial trial of the app highlighted that many users were not aware of the gaze feature typically used in a VR environment, so a tutorial was built into the app to help the user become accustomed to interacting within VR prior to starting their virtual MRI journey for the first time.

We undertook an iterative development cycle of testing to ensure that the app worked on a variety of devices and platforms. For VR, this required additional optimization to ensure that playback across a wide range of devices did not fall below 60 frames per second, where it is recognized that VR-based nausea can be experienced. Furthermore, to ensure the app supported devices across a range of technical specifications, the playback resolution of the video clip was downgraded from the original 4K resolution to 1920x960. This resulted in minimally reduced video clarity and sharpness but with the benefit of decreased download file size (from 480 MB to 161 MB). We found that this final build size was an important consideration because users often have limited storage capacity on their devices. To overcome this, a version of the app was created with the videos being streamed from a remote server rather than being embedded in the app. This was found to reduce the app size to 22 MB. However, to successfully play back the videos, a fast internet connection is required. We found the availability and reliability of such a network in a hospital setting to be limited at times, and given the additional costs of hosting a server from which to stream the videos, we decided not to release the streaming version of the app.

### The Preparation Book

The preparation book was developed to support the app and allowed for closer interaction between the child and health play specialists, radiographers, and parents and to provide a choice of resource for the patient (Multimedia Appendix 1). It contained photos highlighting the same parts of the MRI journey as the app, maintaining consistency of persons acting as radiographers and parents. The electronic version of the preparation book contained hyperlinks to load the 360° videos of the MRI journey that were hosted on YouTube. When displayed using a mobile phone, the videos could be loaded directly from the preparation book and displayed in a Google Cardboard headset using the YouTube Cardboard functionality.

This preparation book was developed in Word (Microsoft Corporation) and exported to a PDF file format for easy distribution and better display on mobile and tablet devices. A shortened version was also developed for specific use within the radiology department to prepare a child immediately prior to their scan. This omitted the stages of arriving at the reception and the safety screening since it was typically given to patients after these stages were complete.

### Implementation and Evaluation

Within the hospital, mobile devices were used with the Z4 mini-headset (BoboVR), and for home use, a disposable Google Cardboard version 2 headset (Access VR Solutions) was mailed to patients. The BoboVR Z4 mini had a faux leather face-pad making it cleanable and therefore the most suitable for use within a hospital environment where the risk of infection must be controlled. The head straps were removed from the headset such that the patient simply held the headset to their face. Given our resource had a maximum VR exposure time of approximately 5 minutes, simply holding the headset was preferred as this avoided the need for adjustments to the straps and allowed for rapid removal of the headset if the patient wanted to stop the experience (eg, if suffering from VR-based simulation sickness).

The evaluation of the resource was undertaken following the National Institute for Health and Care Excellence Evidence Standards Framework for Digital Health Technologies [19]. The goal of the evaluation was to gather anonymous feedback on the initial implementation helping to inform further enhancements of the resource to improve the patient experience. The evaluation is ongoing as part of a registered clinical audit in accordance with King’s College Hospital standard policies and procedures.

We recruited 23 patients (median age 9 years, range 4 to 12 years) attending the Neuroradiology Department at King’s College Hospital during the period November 2016 to February 2017. Patients voluntarily participated in an anonymous survey and were selected to include those who had never had an awake MRI (19/23) or had had an MRI more than 1 year ago (4/23). An upper age limit was set due to the nature of the resource, which was designed to be appropriate for users up to approximately age 12 years. The lower age range was set by the minimum age for which a child was booked to have an awake MRI during the evaluation period. Since the purpose of the evaluation was to provide initial feedback on the resource, a patient group without medical complexities was chosen (ie, patients were excluded if they suffered from a physical or nonphysical disability such as cerebral palsy or autism, which may inhibit their interaction with the VR environment). Patients were identified through the radiology information system as having an appointment for an upcoming brain or spine MRI. The patient was either mailed a headset for them to trial the resource at home or were provided with the resource immediately prior to their MRI when they arrived for their scan. All patients voluntarily participated in an anonymous questionnaire (see Multimedia Appendix 2) which assessed their experience using the preparation resource with a standard 10-point or 5-point Likert scale.

Ten King’s College Hospital staff were also surveyed including health play specialists (2/10), radiographers (7/10), and a health care assistant (1/10; see Multimedia Appendix 3). Staff were included if they had experience using the preparation resource during the patient evaluation period. The survey questionnaire used 10-point and 5-point Likert scales to evaluate staff experience using the resource in preparing children for an MRI.

The app was provided to 5 patients who were originally considered for an MRI under GA. After allowing the child to experience the app, it was thought that the GA could be avoided, and in these patients an awake MRI was attempted. All subsequent images were then evaluated for patient motion artifact.

## Results

Versions of the preparation resource have been created for the MRI journeys for three UK hospitals including King’s College Hospital, The Royal Belfast Hospital for Sick Children, and NHS Highland, freely available under the titles “My MRI at King’s,” “Virtual Reality MRI,” and “My MRI at Raigmore,” respectively.

**Figure 2:**
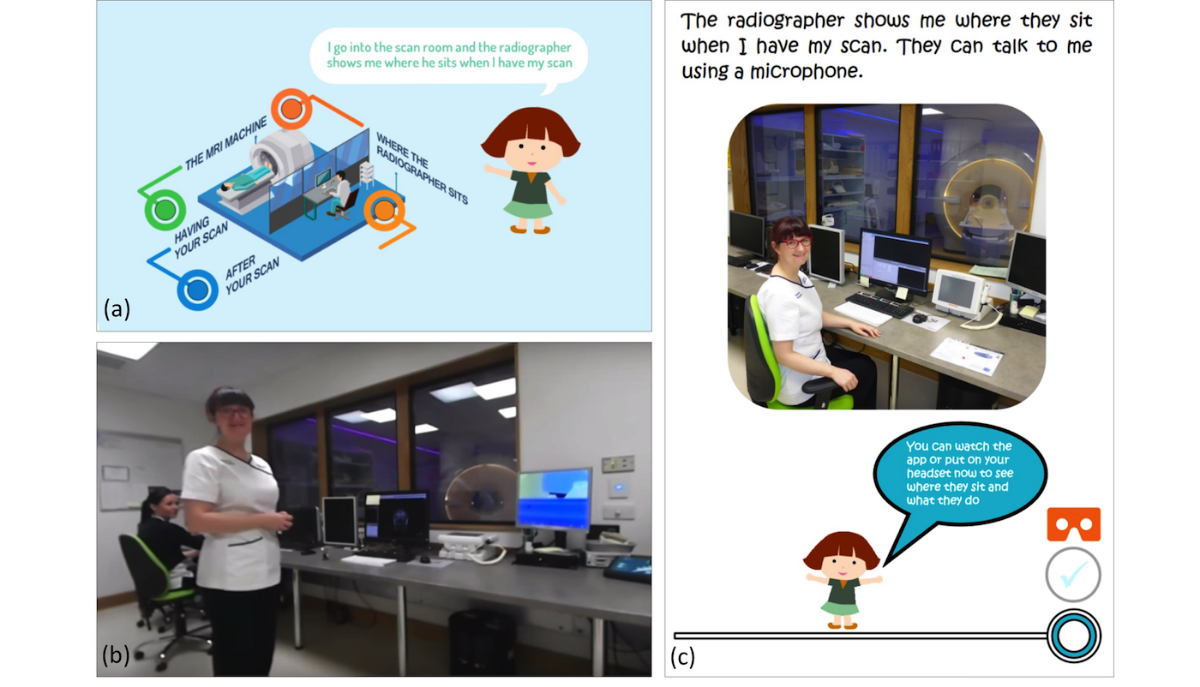
Screen shots from the 'Where the Radiographer Sits' stage of the MRI preparation resource: (a) app cartoon interface, (b) corresponding 360° video, and (c) corresponding page from the preparation book.

**Figure 3:**
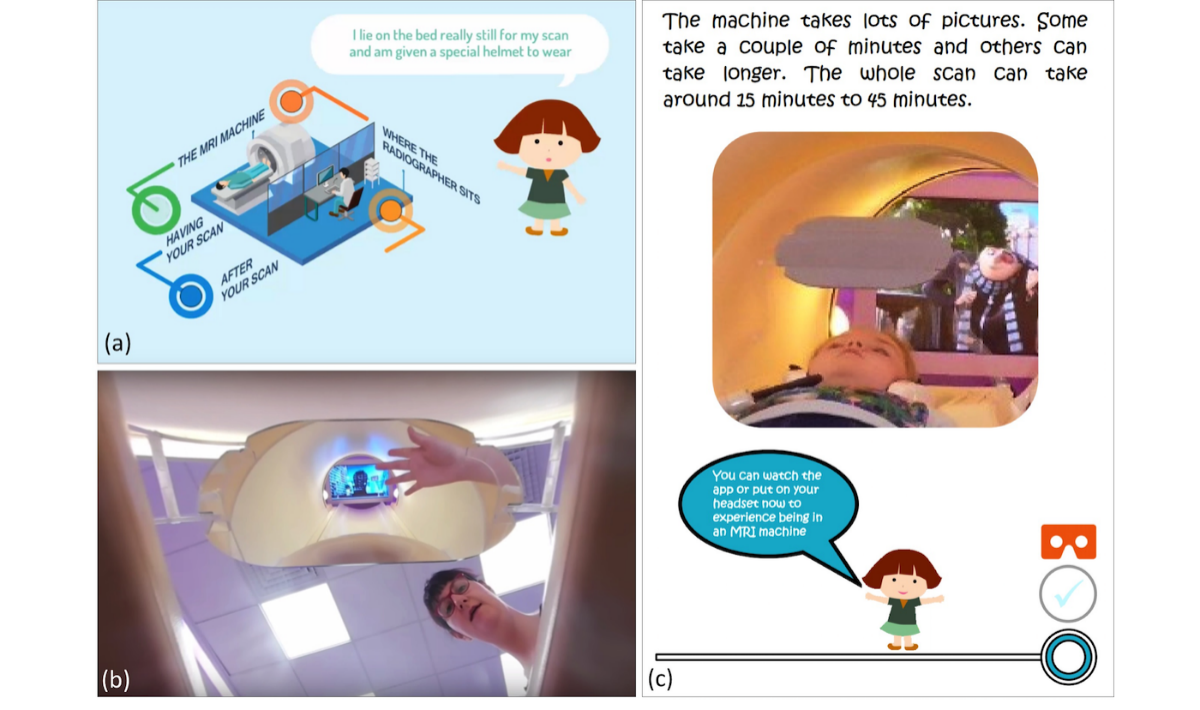
Screen shots from the 'Lying Down, Having Your Scan' stage of the MRI preparation resource: (a) app cartoon interface, (b) corresponding 360° video, and (c) corresponding page from the preparation book.

All versions have the same interface/template and differ only in the embedded 360° videos or photos, which are specific to each hospital site. Sample images are shown in Figures 2 and 3 that highlight the cartoon style interactive interface and stills from the 360° videos. Also shown are the corresponding sections from the preparation book.

The results of the patient questionnaire are shown in Multimedia Appendix 4, which highlights a positive response to the preparation resource. An unexpected outcome of the patient feedback was the impact the app had on parents, several of whom commented on the feedback forms that the app allowed them to better understand their child’s upcoming MRI, helping to reduce their own anxieties and enabling them to better prepare their child.

The results of the staff questionnaire appear to highlight that staff members believe the preparation resource to be a useful tool (Multimedia Appendix 5). All ten staff members surveyed answered questions 1 through 8, while question 9 was not answered by health play specialists since they have limited involvement in directly scanning patients.

Of the 5 patients originally booked for MRI under GA, 4 were able to tolerate an awake MRI. In one case, the child’s anxiety levels prohibited them from entering the scan room, and GA had to be administered. In all cases, no movement was noted by the scanning radiographer or suggested in the radiology reports.

## Discussion

### Principal Findings

In this paper we report the technical aspects involved in creating our preparation resource that uses 360° video footage displayed via an inexpensive VR headset to create an immersive virtual MRI scan experience. Our evaluation highlights both patients and staff responding positively to the resource and the potential it has as a tool to reduce the need for GA.

While acquiring the 360° footage, we experienced some of the well-known pitfalls of creating VR content, many of which are previously described in a similar use of VR to prepare patients for anesthetics procedures [15]. This includes inadvertently inducing feelings of nausea in the viewer by acceleration in the VR environment. Although not fully understood and with a variety of causes, VR sickness can be triggered by conflicting sensory inputs from the visual system, vestibular system, and nonvestibular proprioceptors [20]. Additionally, there is a need to be conscious of filming close to objects and people—the VR equivalent to invading personal space.

These issues are well described in the VR literature and are key aspects to consider if a VR experience is to feel immersive and fulfill the place and plausibility illusion [21]. In a practical sense, when obtaining 360^o^ footage we found it important to undertake quality control of the content. This was done by immediately viewing the footage within a VR headset so we could assess for any VR-specific issues and correct them immediately rather than realizing these issues at a later date.

There has been limited research involving the use of VR in children, and concerns remain regarding its safe use. These concerns include the potential for physical harm, since VR encourages the user to undertake movements while being blinded to the actual physical environment; VR-based simulation sickness [22]; and the potential effect VR has on the child’s vision and balance [23]. To address these concerns, the use of VR equipment often comes with an age restriction from the manufacturer. In the case of the equipment used in this work (Google Cardboard-based headsets), the manufacturer suggests their headset “can be used by children but under adult supervision.” For our purposes, the time spent in VR was limited to approximately 5 minutes (the length of the resource). This is similar to the maximum exposure introduced by other research groups aiming to protect younger children from any adverse effects [24]. These risks were documented in a risk assessment and explained to the parents prior to use. If at any point the patient felt uncomfortable with the experience, the headset could be removed immediately.

We developed two methods to deliver our 360° video content to patients. First, we created an app for free download from the Android Google Play and iOS App Store. We considered deploying to other VR platforms that provide a higher quality and more immersive VR experience (eg, Samsung Gear VR, Google Daydream, Oculus Rift, and HTC Vive environments). However, given the low cost and greater potential for distribution, the Google Cardboard format was in our view the best choice, enabling hospital workers and patients’ families to easily download and use the app with no previous experience with VR. Furthermore, deployment to Android and iOS enables the app to be viewed in tablet mode, which can be useful for patients who do not have a Google Cardboard headset or cannot tolerate the VR environment. Examples include postoperative patients or patients with physical disabilities who cannot easily move.

In younger patients and where there was support from trained clinical staff, the preparation book enabled a closer interaction between staff and child, something that is particularly important for children with high levels of anxiety associated with MRI. Preparation books are widely used by health play specialists in hospitals, and children respond well to seeing visual images and having age-appropriate information that allows them to understand and process what will be happening to them. The preparation book presented here aimed to be an interactive resource. The electronic version linked directly to the 360° videos for viewing within a VR headset. By offering a selection of preparation resources (eg, app or preparation book), choices are provided and individual learning needs for patients of different ages are better met. The need for age-appropriate resources has previously been noted in the literature [7].

Patient and staff surveys highlighted the potential benefit of the preparation resource as a source of information for relieving patient anxieties but also as an enjoyable experience for the child. This enjoyment could be in part due to the novelty factor of VR, where the child may have engaged more with the content due to them experiencing the technology for the first time. In our evaluation we did not assess for any previous exposure to VR or 360° video content, and therefore as the technology becomes ubiquitous, there is potential for our resource to be less engaging and less effective. Literature, however, shows that traditional resources such as preparation booklets and mock scanners reduce patient anxiety in MRI, and numerous studies have measured the benefits of such preparation with patient questionnaires, heart rate measurements [25], or blood prolactin and cortisol levels after the scan [26]. In one study, however, it was found that such preparation had potential to increase anxieties [27].

The only negative patient feedback was from a 12-year-old who felt the app was too babyish. Similarly, the staff survey suggested from their experience the applicable maximum age for the resource was 11 years. On a positive note, the staff survey highlighted that in some cases staff felt the preparation resource potentially reduced patient motion and scan time due to increased compliance. A similar result has been previously reported in a prospective controlled study where patient preparation resources have shown significant reductions in patient motion [5]. The advantage of our resource is that it can be used at any location in the hospital, and parents can use the resource to prepare their child at home.

### Limitations

The purpose of our survey was to provide feedback on our preparation resource to improve and further enhance the patient experience. We did not consider a research-based approach involving, for example, a control group, and so our results are not generalizable without further related work. Likewise, no control group was considered when we applied our resource to the patient case studies who were booked for GA, as our focus was on evaluating the potential for the app to avoid GA. The benefits of preparation resources have previously been investigated in controlled studies that have concluded such interventions can successfully obviate the need for patient sedation [8,28]. In future work, we aim to follow a similar methodology using our VR-based preparation and considering anxiety scores, GA rates, and the presence of motion artifact in images for patients who have been prepared using our VR resource compared to traditional preparation techniques such as preparation booklets and pamphlets.

### Conclusion

The VR preparation resource presented in this article is a novel tool for hospital staff and parents to relieve anxieties of pediatric patients and potentially increase awake MRI scan compliance. The resource is freely available for download on multiple platforms and as such could easily be used by any site scanning children. The method developed could be recreated by others with little effort and has the potential to be expanded to other patient journeys. Our initial experiences using the resource provided nearly unanimous positive feedback, and it was shown for some patients that it potentially helped avoid the need for GA while undergoing MRI.

## Appendix

Multimedia Appendix 1 Preparation book.

Multimedia Appendix 2 Patient questionnaire.

Multimedia Appendix 3 Staff questionnaire.

Multimedia Appendix 4 Patient survey results.

Multimedia Appendix 5 Staff survey results.

## Abbreviations

GA: general anesthesia
MRI: magnetic resonance imaging
MRSE: Magnetic Resonance Safety Expert
NHS: National Health Service
VR: virtual reality
