# Supplementary material for: A Free Virtual Reality Experience to Prepare Pediatric Patients for Magnetic Resonance Imaging: Cross-Sectional Questionnaire Study
Source: JMIR Pediatr Parent. 2019 Apr 18;2(1):e11684. doi: 10.2196/11684 (PMC6716438; doi:10.2196/11684)
Supplement: Multimedia Appendix 1 [file pediatrics_v2i1e11684_app1.pdf]

My MRI Journey

All about Me

You can use this book by itself or with the app or headset- the choice is yours!

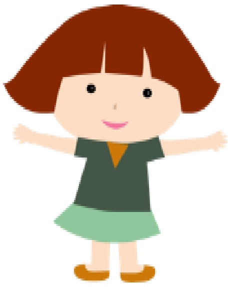

How to use this book

- On the bottom of each page in this book there is a small box, you can tick this box to explain your hospital journey.

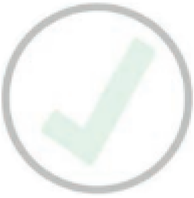

- When you see this symbol you can put on the headset or look at the app to watch a video

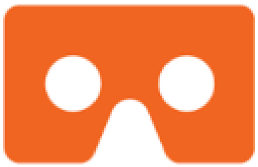

- Fill in the gaps as you go along to tell your story.

- You can keep this book to take home and share with your friends and family.

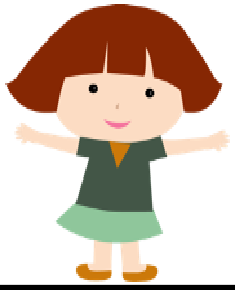

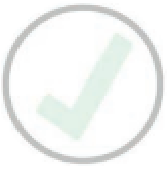

Hello, my name is: ______________________________

I am at the hospital because I need to have an MRI.
This is a book about my visit to Raigmore Hospital.

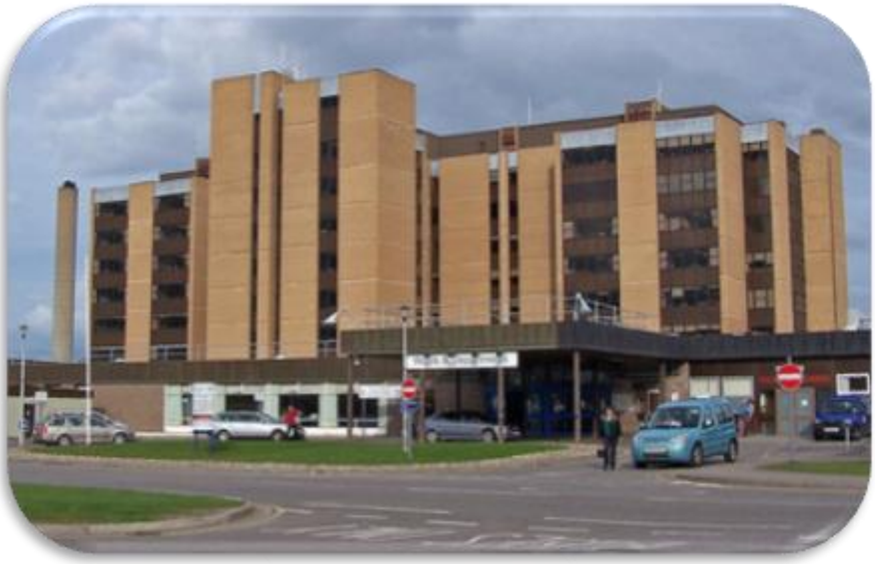

The Hospital

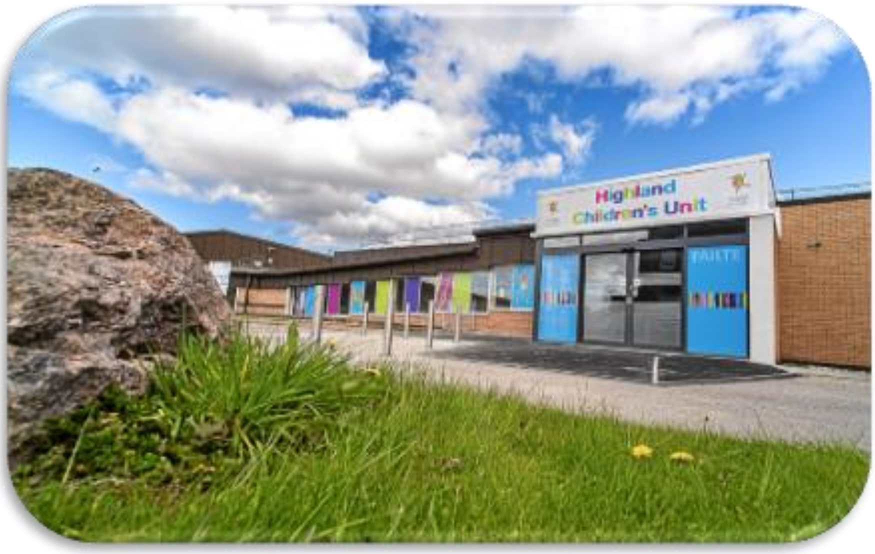

The Children's unit

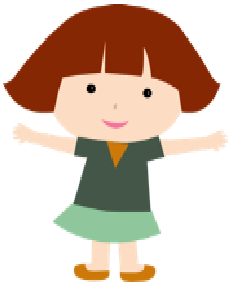

Before visiting today I met the play specialist, I was told an MRI is a type of scan that takes pictures of the inside of my body and it doesn't hurt. I got to visit and look at the MRI machine. The doctors look at the pictures to find out why I am not feeling well.

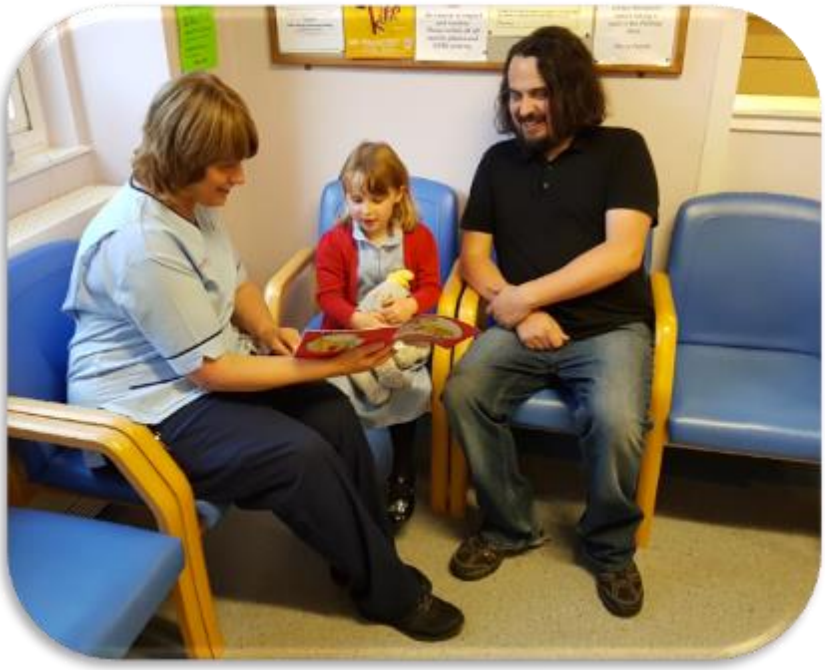

Not everyone meets the play specialist and sometimes they send information to your home so you can see what the scan will be like before you come to hospital, my job is to stay very still in the scan so the picture is clear and not fuzzy.

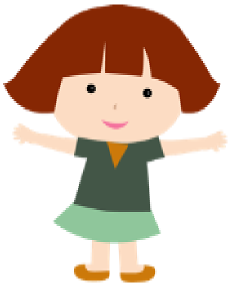

MRI stands for Magnetic Resonance Imaging, the machine makes loud sounds when it is working and this is the coils inside the machine being turned on and off.

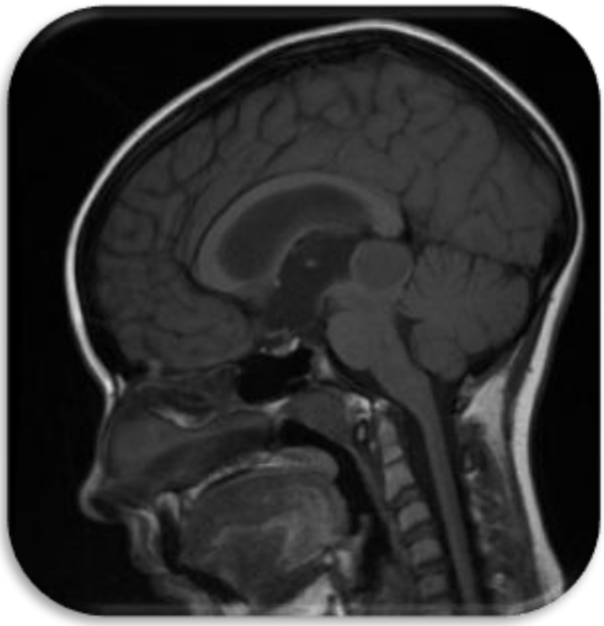

Can you guess what these picture are of?

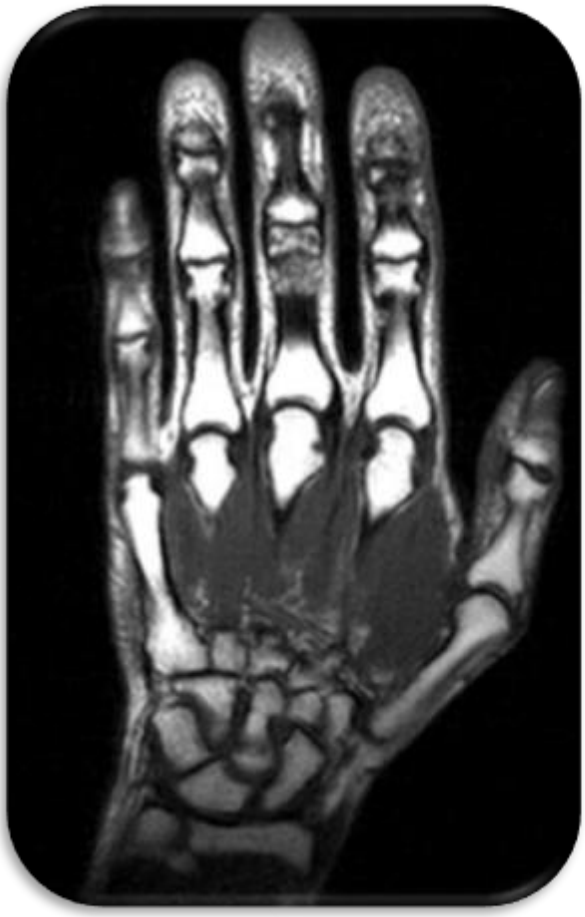

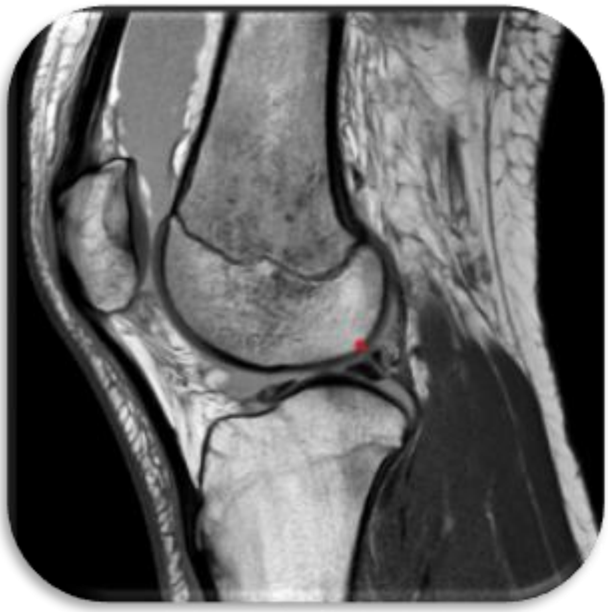

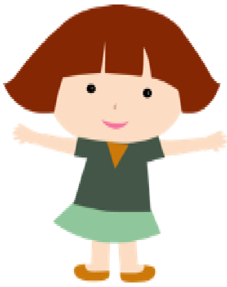

When I get to the hospital I visit the MRI department, some patients come to the department from the children's wards but I am just visiting today for my appointment.

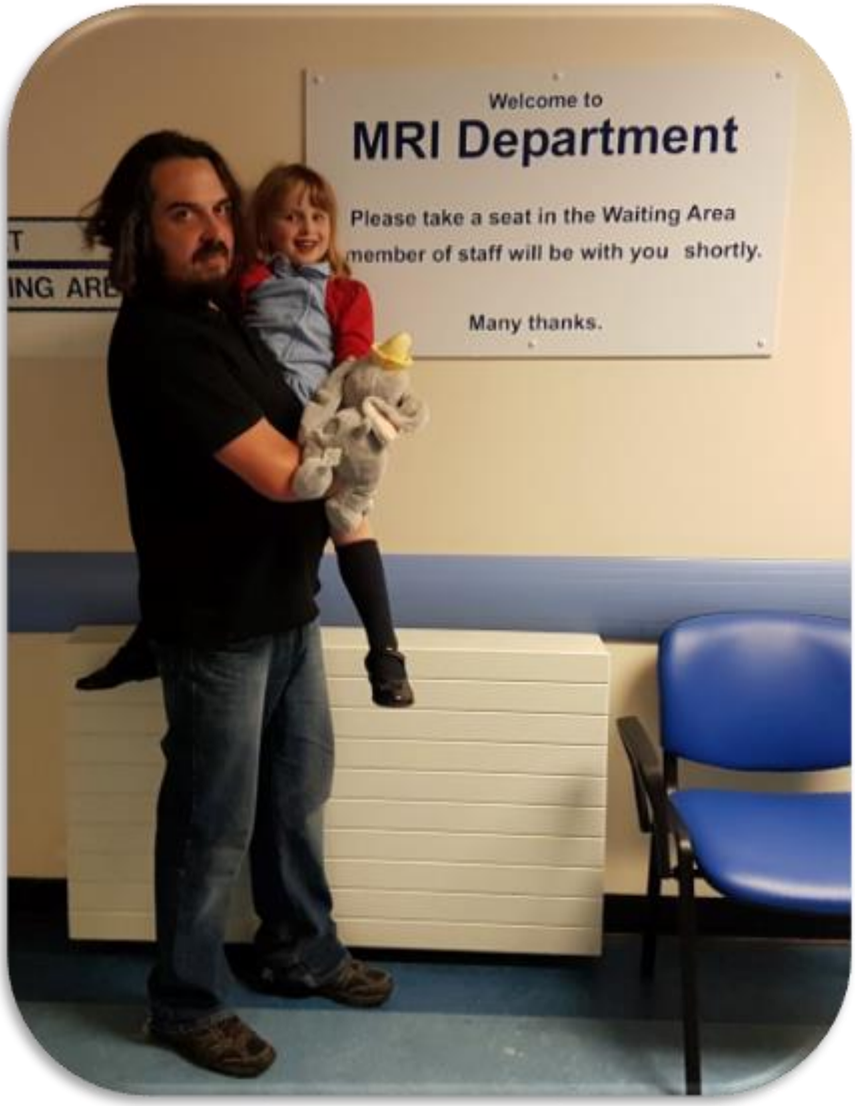

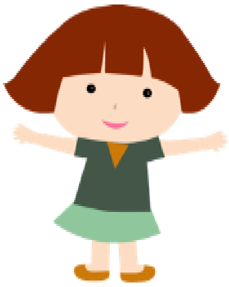

When I arrived at the MRI department I got to play in the reception area until my name is called.

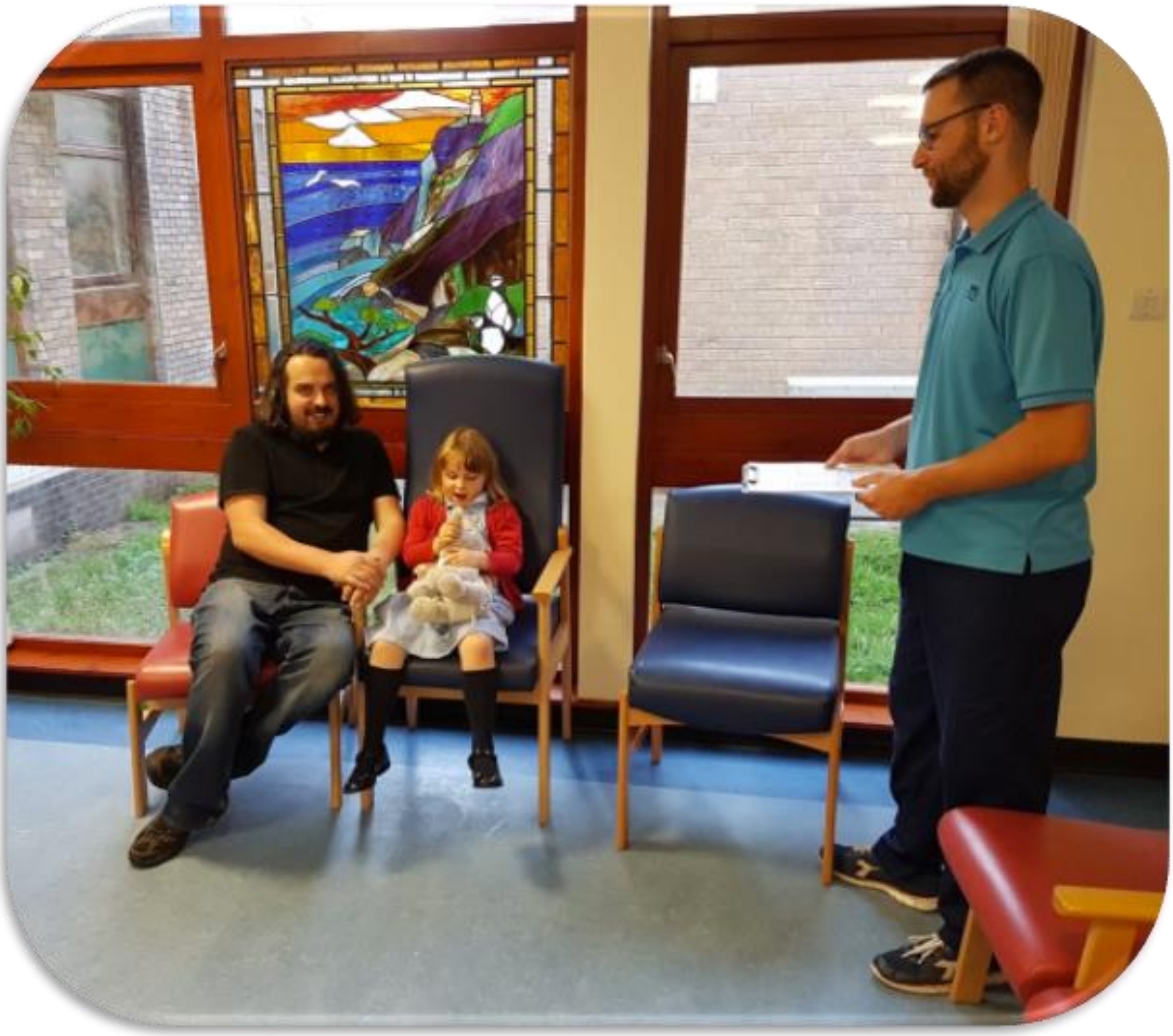

Tap or click on me to see the waiting area

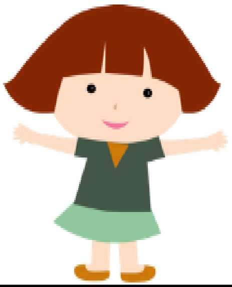

We go to another room where they ask me questions and we fill in a special checklist together.

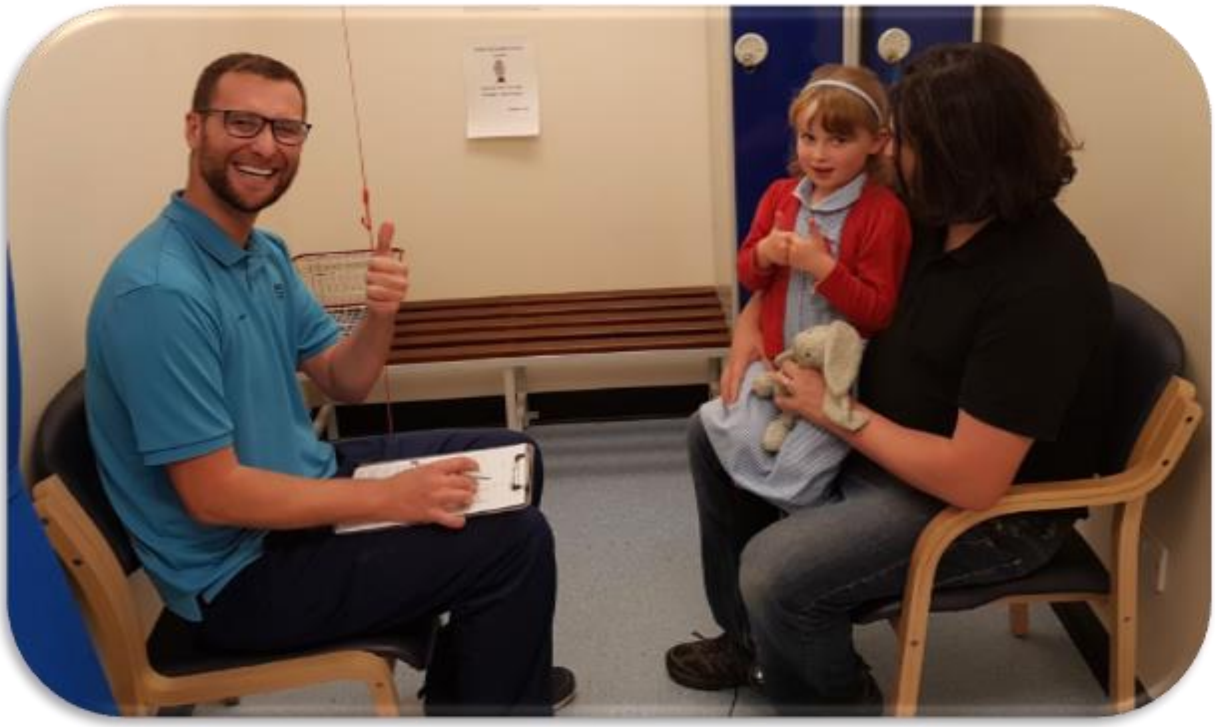

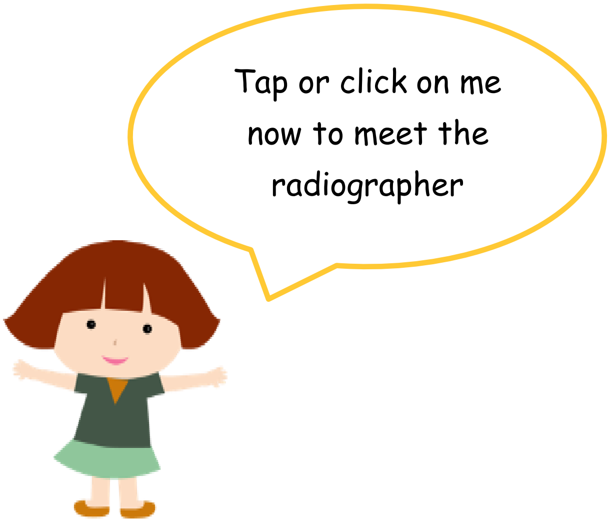

The radiographer told me I can't have anything with metal on in the scan; this can be things like phones, keys, earrings and hair clips.

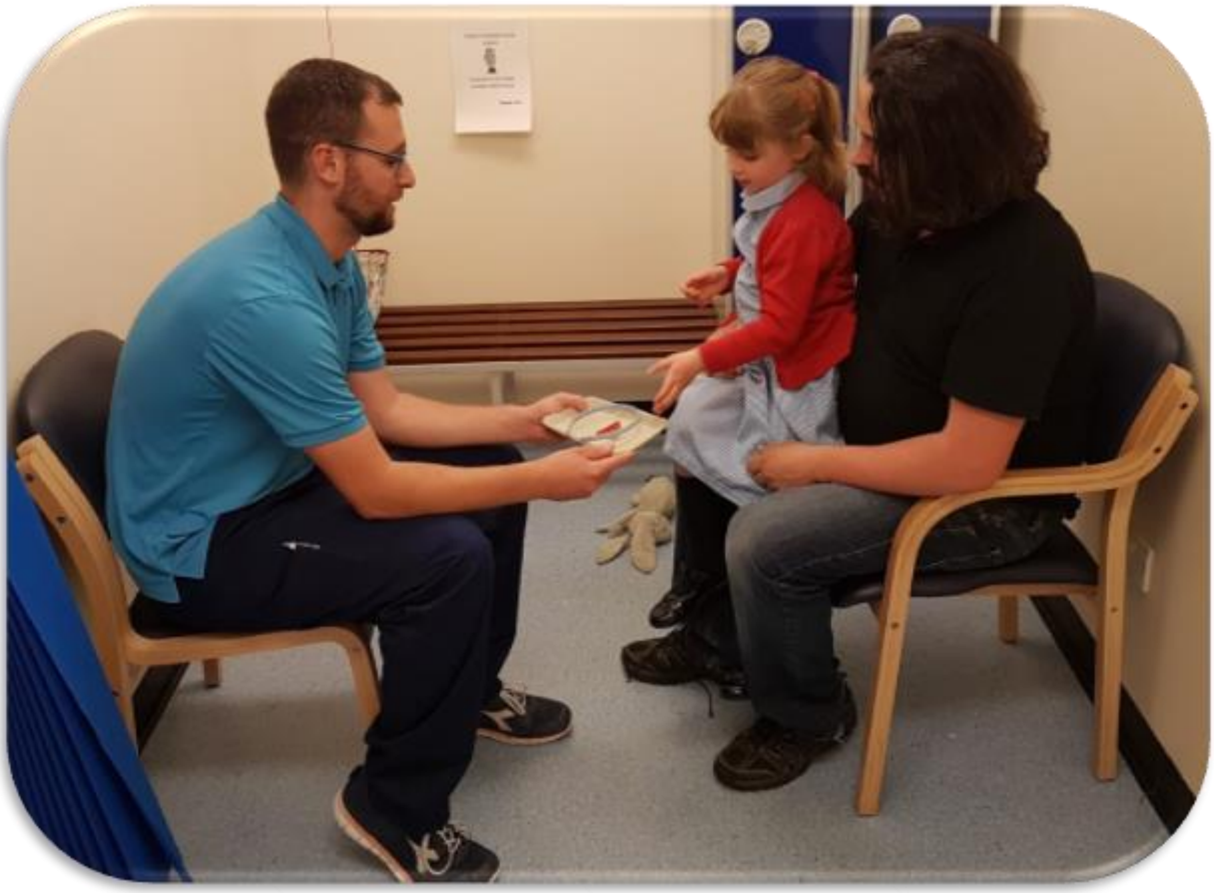

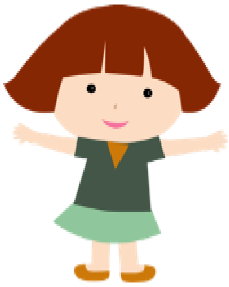

The radiographer shows me where he sits and the buttons he pushes to take the pictures. He can talk to me using a microphone during the scan.

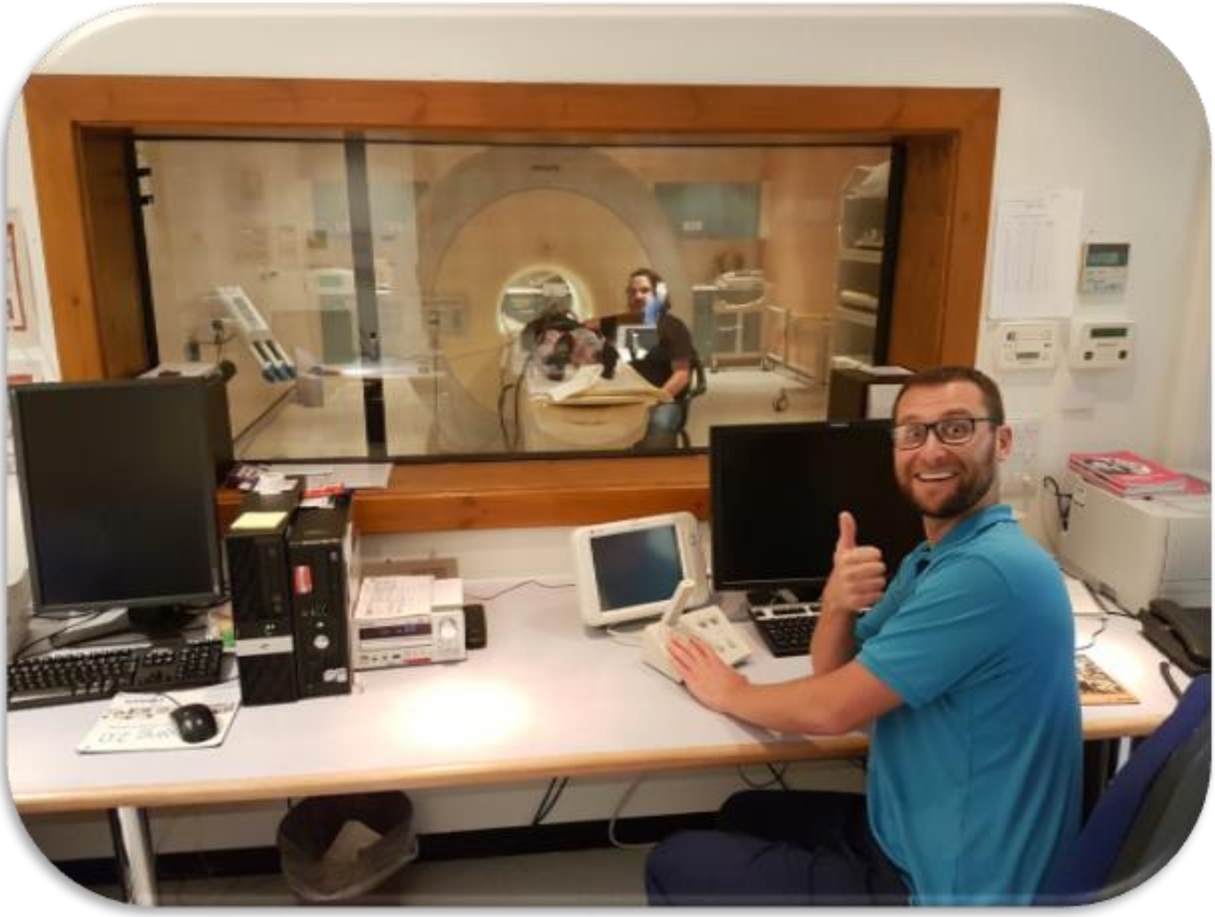

Tap or click on me now to see where they sit and what they do

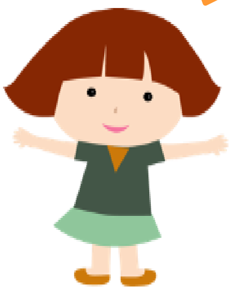

The radiographer shows me the machine that's like a short tunnel that's open at both ends, it's noisy and this is the machine working.

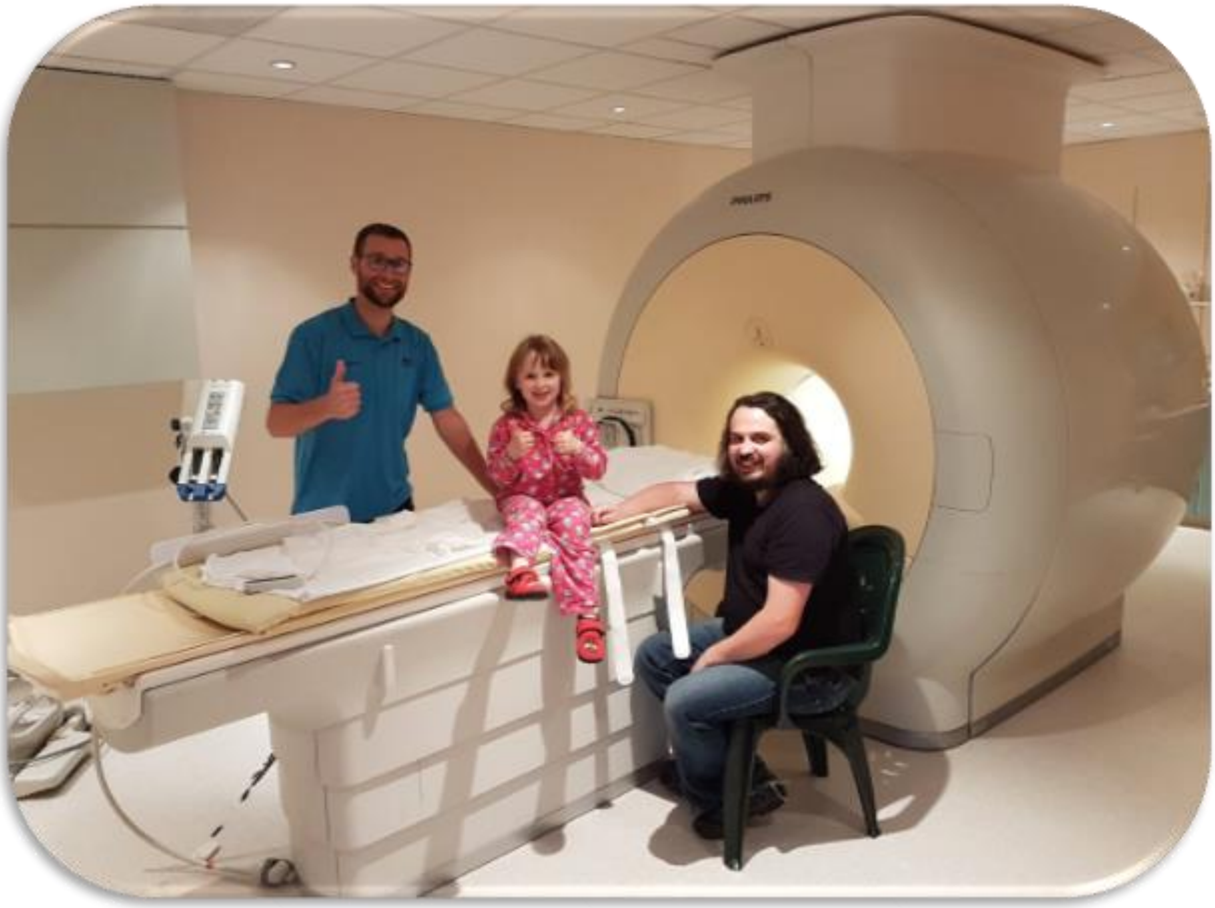

Tap or click on me now to see the MRI machine

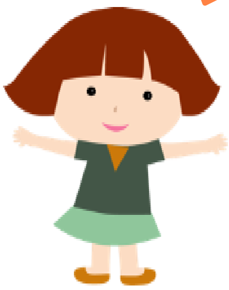

I go in to the MRI room with the radiographer who tells me what will happen; I lie on a bed that moves up and down and in and out of the tunnel.

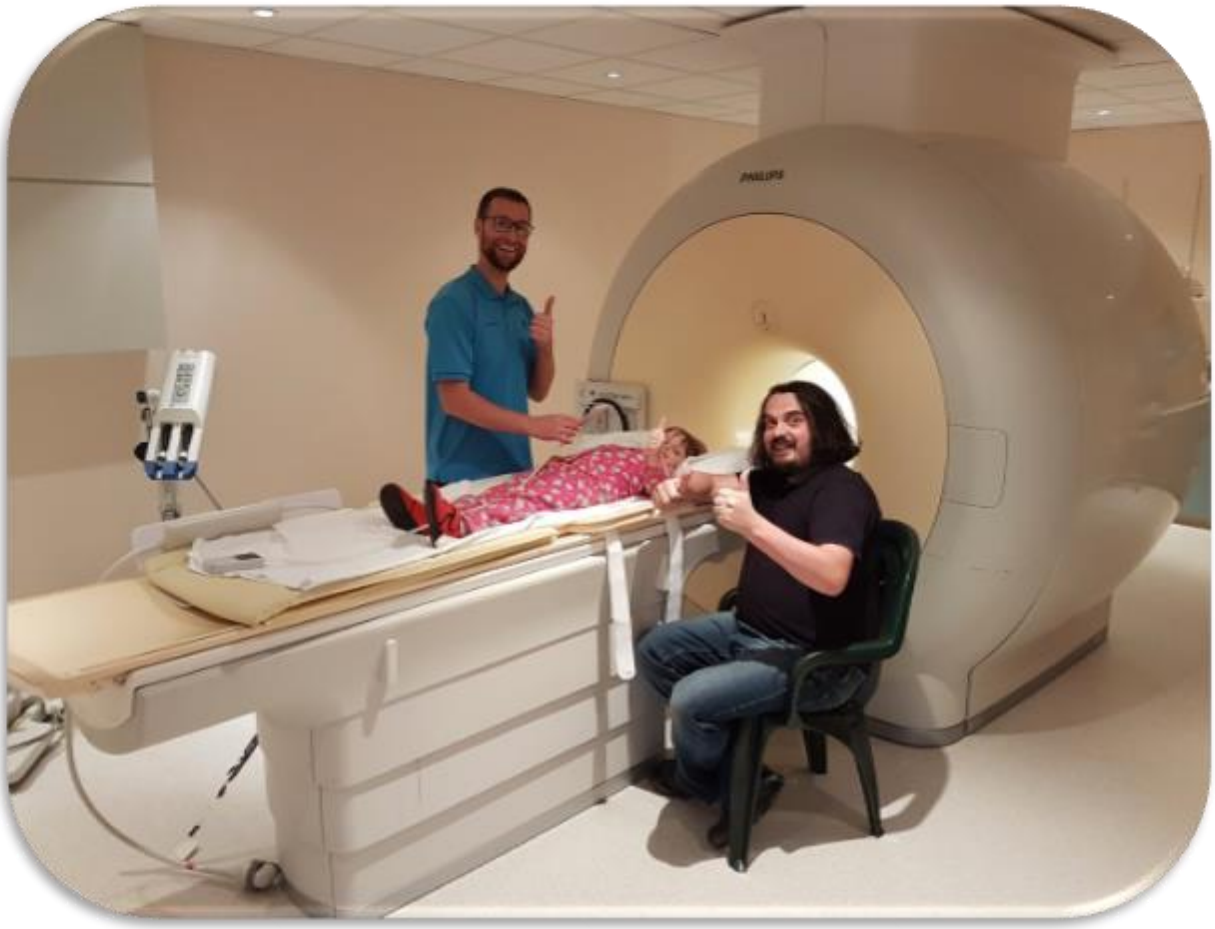

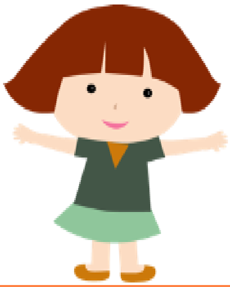

I am given headphones to wear because the machine is noisy, I can still hear some of the sounds though.

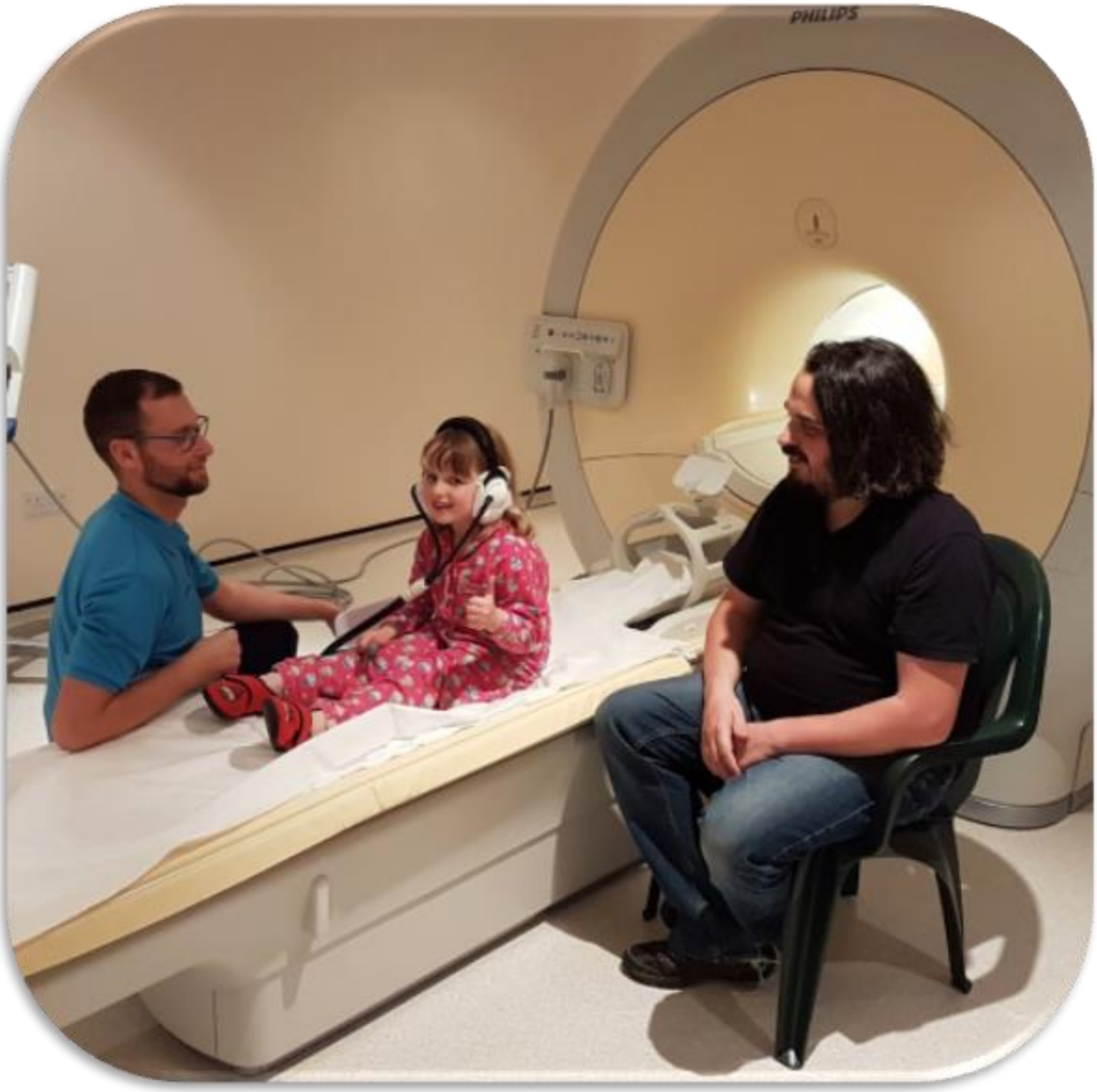

Sometimes I am given a special helmet to wear because they are taking a picture of my head. It has a mirror on so I can see around the room.

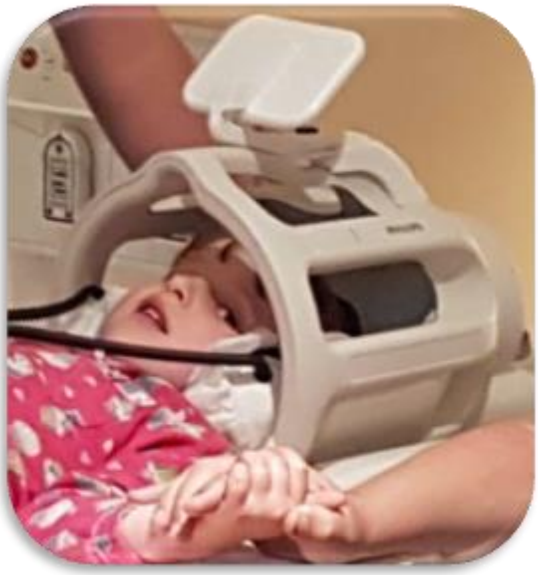

Sometimes I am given a special blanket because they are taking a picture of my body

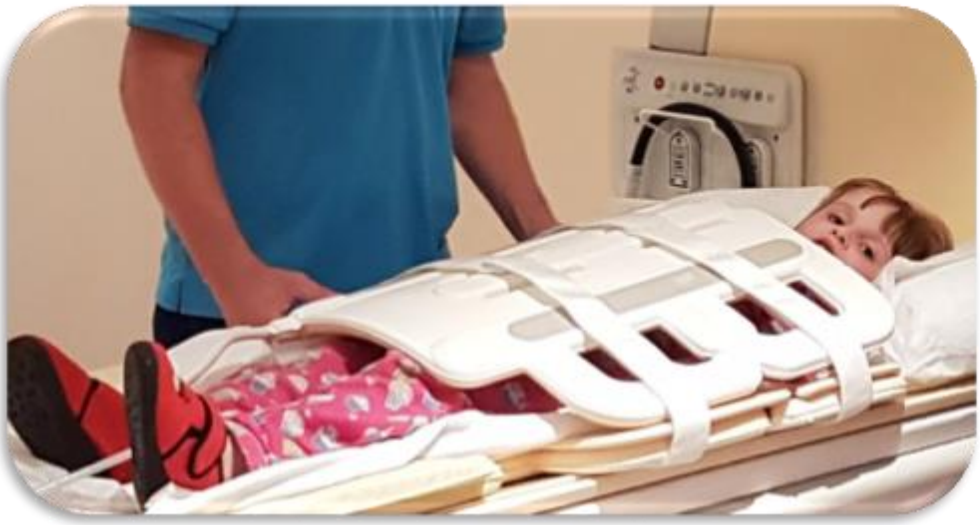

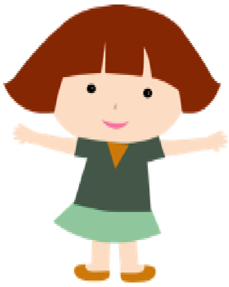

Lots of pictures are taken and some take a couple of minutes and others can be longer. The scan can be around 15 minutes to 45 minutes.

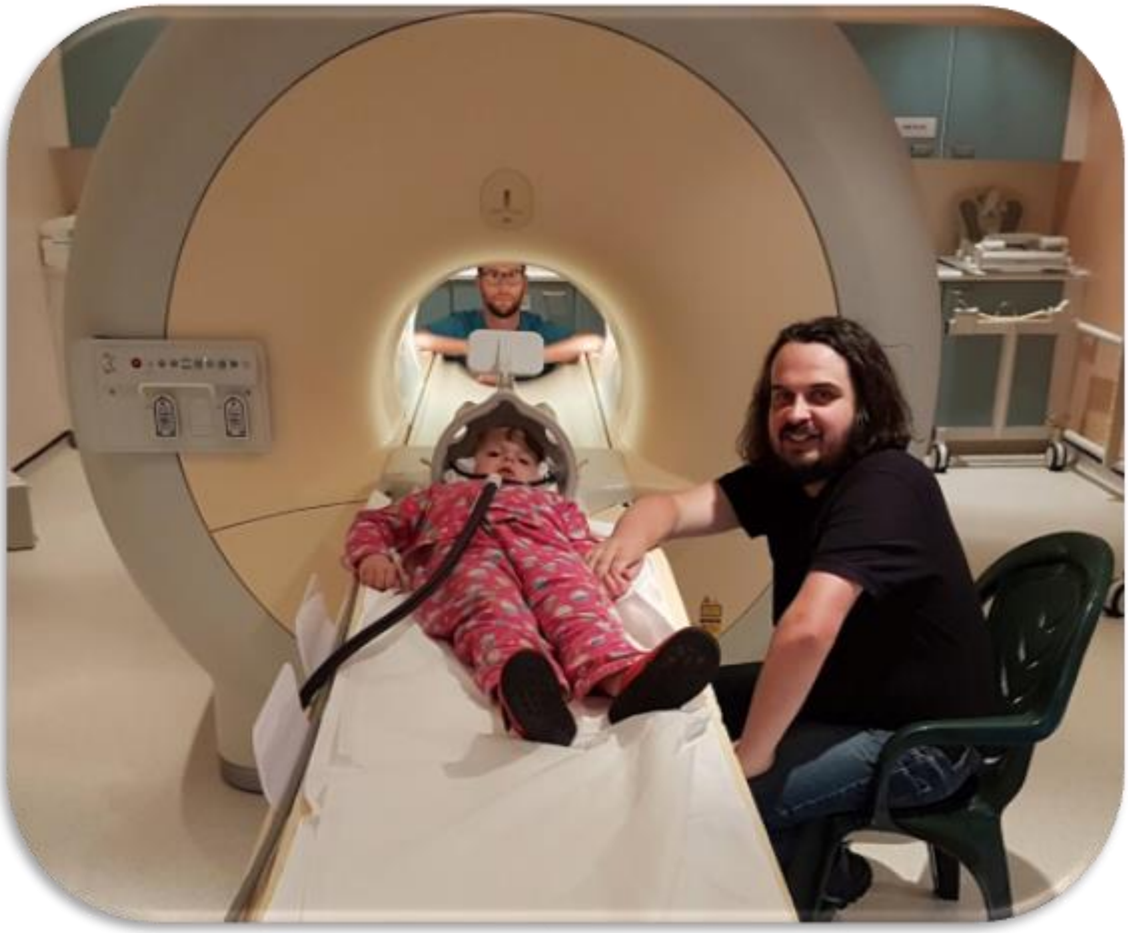

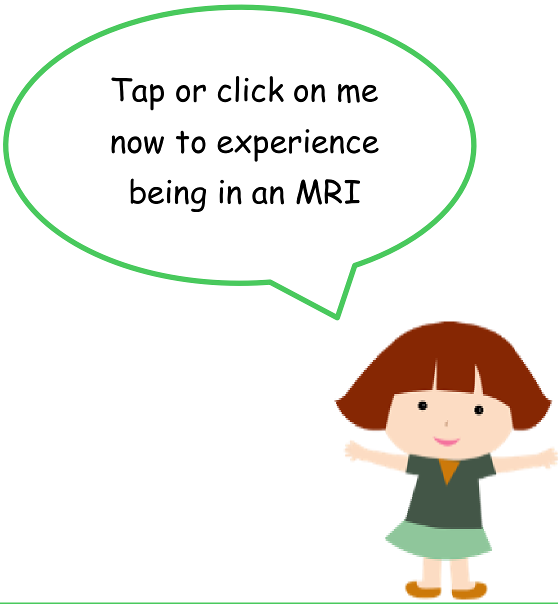

I had my ______________________________ stay with me in the room when I had my scan.

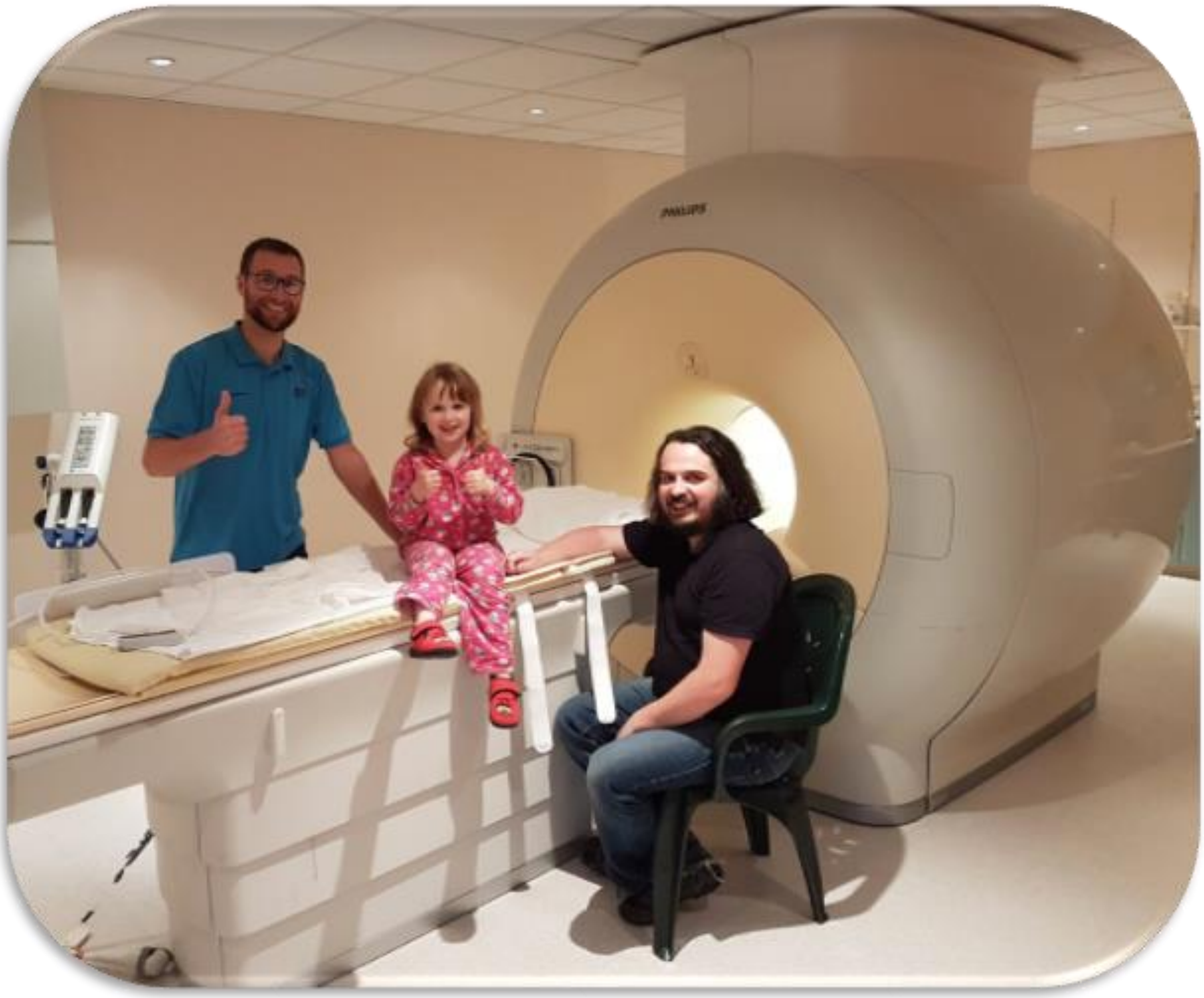

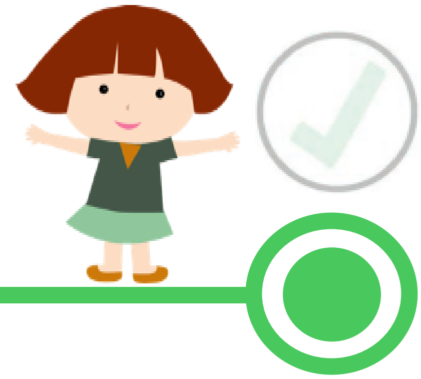

When I am finished the radiographer takes my helmet off and I give back the ear plugs and headphones. I can now go home; some children go back to the ward they are staying on.

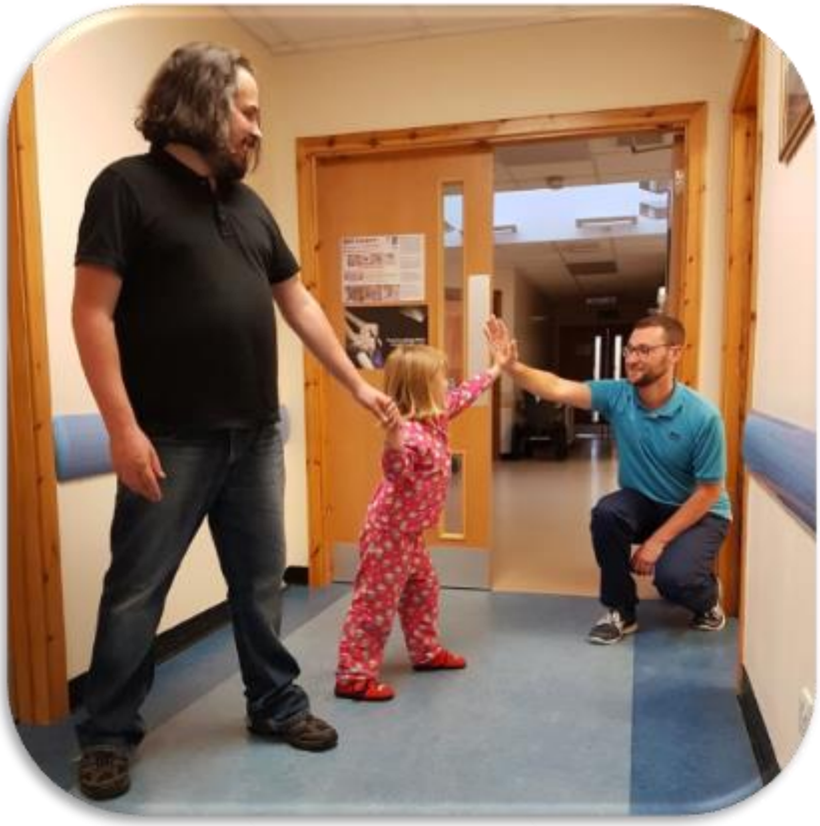

I won't get my results right away

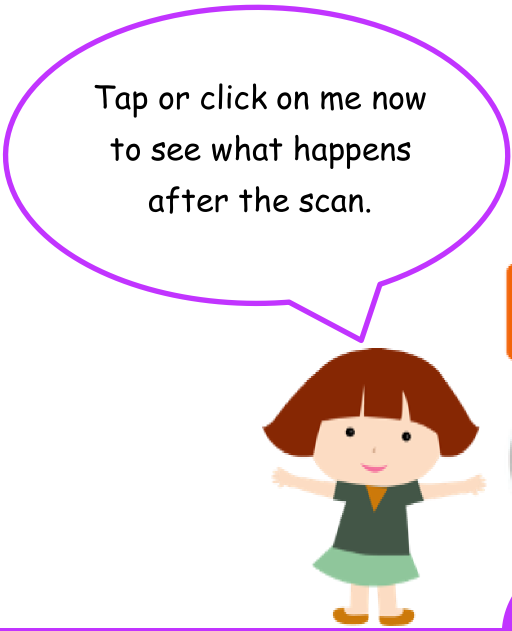

My Notes/ feelings and thoughts
