# Supplementary material for: A Free Virtual Reality Experience to Prepare Pediatric Patients for Magnetic Resonance Imaging: Cross-Sectional Questionnaire Study
Source: JMIR Pediatr Parent. 2019 Apr 18;2(1):e11684. doi: 10.2196/11684 (PMC6716438; doi:10.2196/11684)
Supplement: Multimedia Appendix 2 [file pediatrics_v2i1e11684_app2.pdf]

Questionnaire – Evaluation

This questionnaire is being used to collect information around preparation tools for children and young people, the data will be accessible by medical professionals and the general public. The questionnaire is anonymous and you do not need to put your name on it.

1. **How old is your child?** ☐

2. **How enjoyable did your child find using the app/booklet?**

Not at all — Very

0 1 2 3 4 5 6 7 8 9 10

3. **How helpful did your child find the app/booklet?**

Not at all — Very

0 1 2 3 4 5 6 7 8 9 10

4. **How easy to use did your child find the app/booklet?**

Not at all — Very

0 1 2 3 4 5 6 7 8 9 10

5. **The app/booklet answered my child's thoughts/questions about having an MRI**

1	2	3	4	5
Strongly Disagree	Disagree	Neither agree or disagree	Agree	Strongly Agree

6. **After using the app/booklet my child feels more positive about having an MRI**

1	2	3	4	5
Strongly Disagree	Disagree	Neither agree or disagree	Agree	Strongly Agree

7. **Would you recommend the app/booklet to other young people?**

Yes ☐ No ☐

8. **Any other comments/suggestions**

Thank you for filling in this questionnaire – could you please bring it back and hand to the receptionist on the day of your child's MRI appointment.
