# Supplementary material for: A Free Virtual Reality Experience to Prepare Pediatric Patients for Magnetic Resonance Imaging: Cross-Sectional Questionnaire Study
Source: JMIR Pediatr Parent. 2019 Apr 18;2(1):e11684. doi: 10.2196/11684 (PMC6716438; doi:10.2196/11684)
Supplement: Multimedia Appendix 3 [file pediatrics_v2i1e11684_app3.pdf]

Questionnaire – Staff

This questionnaire is being used to collect information around staff experiences of using preparation tools for children and young people, the data will be accessible by medical professionals and the general public. The questionnaire is anonymous and you do not need to put your name on it.

1. What age range do you think the app/booklet is appropriate for (please circle all)

5 6 7 8 9 10 11 12 13 14 15

2. How enjoyable do you think children find the app/booklet?

Not at all										Very
0	1	2	3	4	5	6	7	8	9	10

3. How helpful did you think children find the app/booklet?

Not at all										Very
0	1	2	3	4	5	6	7	8	9	10

4. How easy to use do you think children find the app/booklet?

Not at all										Very
0	1	2	3	4	5	6	7	8	9	10

5. Do you think the app/booklet answers a child's thoughts/questions about having an MRI

1	2	3	4	5
Strongly Disagree	Disagree	Neither agree or disagree	Agree	Strongly agree

6. Do you think children are more positive about having an MRI after using the app/booklet?

1	2	3	4	5
Strongly Disagree	Disagree	Neither agree or disagree	Agree	Strongly agree

7. Would you recommend the app/booklet for children to use prior to their MRI?

Yes ☐ No ☐

8. **Have you found the app/booklet useful in your job role of preparing children for an MRI or scanning children?**

1	2	3	4	5
Strongly Disagree	Disagree	Neither agree or disagree	Agree	Strongly agree

9. **The app/booklet has been designed with the aim to improve patient experience of MRI. Please tick if you feel the following aims have been achieved OR What aspects of the app/booklet have you find useful?**

- After using the app/booklet children appear more at ease prior to their MRI
- After using the app/booklet Children better understand what will happen for their MRI
- After using the app/booklet children seem better at laying still for their MRI
- The app/booklet can save time when preparing children for their MRI
- The app/booklet can help minimize the scan time for the child's MRI (e.g. from reduced movement or shorter scanner preparation times)
- The app/booklet can save a child requiring a GA for their scan
- Other

Please describe ____

10. **Are there any areas of the app/booklet that you feel should be changed or improved?**

11. **What is your job role? (please circle)**

Play specialist   Anaesthetics nurse   Radiographer   Health care assistant

12. **Any other comments/suggestions**
