# Supplementary material for: A Free Virtual Reality Experience to Prepare Pediatric Patients for Magnetic Resonance Imaging: Cross-Sectional Questionnaire Study
Source: JMIR Pediatr Parent. 2019 Apr 18;2(1):e11684. doi: 10.2196/11684 (PMC6716438; doi:10.2196/11684)
Supplement: Multimedia Appendix 4 [file pediatrics_v2i1e11684_app4.pdf]

**Question**	**Median response**
1. How old is your child?	9
2. How enjoyable did your child find using the app/booklet?	8.5
3. How helpful did your child find the app/booklet?	8
4. How easy to use did your child find the app/booklet?	10
5. The app/booklet answered my child's thoughts/questions about having an MRI	Agree
6. After using the app/booklet my child feels more positive about having an MRI	Agree
7. Would you recommend the app/booklet to other young people?	100% Yes
