# Supplementary material for: A Free Virtual Reality Experience to Prepare Pediatric Patients for Magnetic Resonance Imaging: Cross-Sectional Questionnaire Study
Source: JMIR Pediatr Parent. 2019 Apr 18;2(1):e11684. doi: 10.2196/11684 (PMC6716438; doi:10.2196/11684)
Supplement: Multimedia Appendix 5 [file pediatrics_v2i1e11684_app5.pdf]

**Question**	**Median response**
1. What age range do you think the app/booklet is appropriate for (please circle all)	5 - 11.5
2. How enjoyable do you think children find the app/booklet?	8.5
3. How helpful did you think children find the app/booklet?	9
4. How easy to use do you think children find the app/booklet?	9
5. Do you think the app/booklet answers a child's thoughts/questions about having an MRI	Strongly agree
6. Do you think children are more positive about having an MRI after using the app/booklet?	Strongly agree
7. Would you recommend the app/booklet for children to use prior to their MRI?	Yes (100%)
8. Have you found the app/booklet useful in your job role of preparing children for an MRI or scanning children?	Strongly agree
9. The app/booklet has been designed with the aim to improve patient experience of MRI. Please tick if you feel the following aims have been achieved OR What aspects of the app/booklet have you find useful?	**Number of respondents**
After using the app/booklet children appear more at ease prior to their MRI	5 (63%)
After using the app/booklet Children better understand what will happen for their MRI	5 (63%)
After using the app/booklet children seem better at laying still for their MRI	5 (63%)
The app/booklet can save time when preparing children for their MRI	5 (63%)
The app/booklet can help minimize the scan time for the child's MRI (e.g. from reduced movement or shorter scanner preparation times)	5 (63%)
The app/booklet can save a child requiring a GA for their scan	8 (100%)
Other	0
